# Media Data and Vaccine Hesitancy: Scoping Review

**DOI:** 10.2196/37300

**Published:** 2022-08-10

**Authors:** Jason Dean-Chen Yin

**Affiliations:** 1 School of Public Health Li Ka Shing Faculty of Medicine The University of Hong Kong Hong Kong China (Hong Kong)

**Keywords:** review, social media, traditional media, vaccine hesitancy, natural language processing, digital epidemiology

## Abstract

**Background:**

Media studies are important for vaccine hesitancy research, as they analyze how the media shapes risk perceptions and vaccine uptake. Despite the growth in studies in this field owing to advances in computing and language processing and an expanding social media landscape, no study has consolidated the methodological approaches used to study vaccine hesitancy. Synthesizing this information can better structure and set a precedent for this growing subfield of digital epidemiology.

**Objective:**

This review aimed to identify and illustrate the media platforms and methods used to study vaccine hesitancy and how they build or contribute to the study of the media’s influence on vaccine hesitancy and public health.

**Methods:**

This study followed the PRISMA-ScR (Preferred Reporting Items for Systematic Reviews and Meta-Analyses extension for Scoping Reviews) guidelines. A search was conducted on PubMed and Scopus for any studies that used media data (social media or traditional media), had an outcome related to vaccine sentiment (opinion, uptake, hesitancy, acceptance, or stance), were written in English, and were published after 2010. Studies were screened by only 1 reviewer and extracted for media platform, analysis method, the theoretical models used, and outcomes.

**Results:**

In total, 125 studies were included, of which 71 (56.8%) used traditional research methods and 54 (43.2%) used computational methods. Of the traditional methods, most used content analysis (43/71, 61%) and sentiment analysis (21/71, 30%) to analyze the texts. The most common platforms were newspapers, print media, and web-based news. The computational methods mostly used sentiment analysis (31/54, 57%), topic modeling (18/54, 33%), and network analysis (17/54, 31%). Fewer studies used projections (2/54, 4%) and feature extraction (1/54, 2%). The most common platforms were Twitter and Facebook. Theoretically, most studies were weak. The following five major categories of studies arose: antivaccination themes centered on the distrust of institutions, civil liberties, misinformation, conspiracy theories, and vaccine-specific concerns; provaccination themes centered on ensuring vaccine safety using scientific literature; framing being important and health professionals and personal stories having the largest impact on shaping vaccine opinion; the coverage of vaccination-related data mostly identifying negative vaccine content and revealing deeply fractured vaccine communities and echo chambers; and the public reacting to and focusing on certain signals—in particular cases, deaths, and scandals—which suggests a more volatile period for the spread of information.

**Conclusions:**

The heterogeneity in the use of media to study vaccines can be better consolidated through theoretical grounding. Areas of suggested research include understanding how trust in institutions is associated with vaccine uptake, how misinformation and information signaling influence vaccine uptake, and the evaluation of government communications on vaccine rollouts and vaccine-related events. The review ends with a statement that media data analyses, though groundbreaking in approach, should supplement—not supplant—current practices in public health research.

## Introduction

### Media and Public Health

The media are important for public health research. They are a source of information, a broadcasting station, an issue identifier, and a perception molder, among many things. Exposure to the media can thus shape health-related perceptions and, therefore, behaviors. This area of research has extended from the fields of psychology and social psychology and primarily looks at *effects of media* [1]*.* It primarily asks the following question: what are the consequences of media exposure at an individual, group, institutional, and social system level? This question highlights the different levels at which communication occurs.

At an individual (or micro) level, there are three interwoven theoretical areas: expectancy value, information processing, and message effect [1]. Expectancy value theories posit that health behaviors are motivated by beliefs and expectancies regarding an outcome and the values placed on it. Theories such as the health belief model (HBM) [2], theory of planned behavior [3], and theory of reasoned action [4] all account for how media exposure can affect the motivations, attitudes, and behaviors of individuals regarding a decision. Information processing focuses on how psychological processing occurs and leads to either changes or reinforcements in attitude. Examples include the elaboration likelihood model (ELM) [5], extended parallel processing model [6], and protective action decision model [7], which focus on how cues and the environment affect cognitive processes in decision-making, whether this induces a deliberate and thoughtful or passive and peripheral processing of information. These types of studies also focus on how messaging units and the different manifestations (eg, text and images) influence information processing. This alludes to the last theoretical area, message effects, which looks at how the construction of messaging influences information processing [8]. The most common approach in this area is the study of framing, which involves understanding how the media encodes messages through signs and symbols, thereby characterizing an issue and indirectly characterizing how entities should perceive it. These 3 areas, although presented separately, are tightly linked: message effects will affect processing and, thereby, expectations and values placed on outcomes.

At a societal (macro) level, much work has been done on the media’s role in agenda setting. In agenda setting theory, the media can influence the importance of topics to the public and, thus, the topic’s prioritization as a social problem [9]. This process unfurls in two simultaneous steps—framing and amplification. As stated earlier, the construction and characterization of messages shape public perception of the issue. This has a spillover effect of priming the audience to reconsider their evaluation of an outcome of or the value placed on a topic. When the media are broadcast on different channels, they inadvertently amplify those framed signals, highlighting the media’s inherent nature as an amplification station. This concept was captured succinctly in the social amplification of risk framework (SARF) by Kasperson [10], focusing on how topics, events, or hazards interact with psychological, social, institutional, and cultural processes that result in amplification or attenuation of the perception of said topics, events, or hazards. In this process, the media is an institution that acts as an amplification station bringing attention to issues. Amplifying, coupled with framing, shapes public opinion.

Although the schema of micro and macro analyses is separated for presentation, emphasis should be placed on their interconnectedness, especially in a complicated media landscape. The agenda and framing of topics and their subsequent propagation through media channels may shape public and individual opinions. These upstream effects proceed to mold individual processing, expectations, and values around the topic. However, the media, presented as a monolithic concept thus far, can be deconstructed. The growth of alternative social media channels for communication has blurred who or what is considered media. Individual users can act as amplification stations and create content for access on large scales, upending the monopoly traditional media channels had on agenda setting, framing, and amplification. In short, everyone is a purveyor of information. This landscape shapes the mosaic of perceptions of an issue [11]. The next question is then what issue is important for public health?

### Vaccine Hesitancy

The World Health Organization (WHO) listed vaccine hesitancy—a “delay in acceptance or refusal of vaccination despite availability of vaccination services” [12]—as one of the top 10 threats to global health in 2019 [13]. In a paper published by the WHO Strategic Advisory Group of Experts on Immunization, they proposed a matrix of determinants that identified three *categories* of influences—contextual, individual and group, and vaccine-specific—that shape the decision to accept, delay, or outright reject vaccines [12]. Several factors nested within these categories point to the media as potentially influencing vaccine uptake. For example, in contextual influences, “communication and media environment” explicitly highlights media as a contextual influence; the individual and group influence category contains “immunization as a social norm,” which can be shaped by media portrayals; and vaccine-specific issues include the factors “introduction of a new vaccine,” “the strength of recommendation,” and “risk/benefit from scientific evidence,” all of which are potentially shaped by media coverage and portrayal. Thus, the media and vaccine hesitancy are linked.

Although not a new phenomenon, vaccine hesitancy has been brought back into the limelight through 2 developments. The first development is the growth of social media as a platform for information consumption. The capacity of the individual to assume the role of media in information creation and propagation has complicated the information landscape. These complications include the credibility of the news source and the sheer increase in the size of information production. A resulting externality that may influence vaccine hesitancy is the exposure of the public to misinformation, both unintentional and deliberate. Another externality is exposure to the platforms’ algorithms that perpetuate information to reinforce existing beliefs, encouraging polarization (echo chambers). The second development thrusting the vaccine debate to center page is the COVID-19 pandemic. Although SARS-CoV-2 stagnated economies through 2020 and 2021, the vaccine was thought to be the exit strategy. However, this was not without marring public criticism regarding its development, efficacy, side effects, and necessity, among other concerns. Throughout the cycle of new variants and boosters after the initial introductions, vaccine-hesitant speech and behavior continued to propagate. Much of this was fueled on social media, which further amplified messaging.

### Objectives

Alongside the public discussion was the proliferation of academic studies analyzing social media to better understand vaccine hesitancy. This proliferation is due in part to the growing number of media platforms but is also the result of paralleling advances in computing and analysis tools that process and handle big data. To date, there have been no studies that catalog the types of media platforms and analysis methods used to study vaccine hesitancy and if there are any consistent findings. To bridge this gap, the objectives of this study were to answer what platforms are studied and how the data contained are analyzed. The aim of this review was to understand how using these platforms and methods *builds* or *contributes* to the existing knowledge of the literature on the media’s influence on vaccine hesitancy and, thereby, public health.

## Methods

### Overview

This review summarized studies on vaccine hesitancy using any form of *media data*—a catchall term for traditional and social media. Traditional media are loosely defined as any media before the advent of digital media. This review followed the guidelines proposed by Arskey and O’Malley [14] and the Joanna Briggs Institute [15]. All reporting of findings is in accordance with the guidelines specified by the PRISMA-ScR (Preferred Reporting Items for Systematic Reviews and Meta-Analyses extension for Scoping Reviews; Multimedia Appendix 1) [16]. The protocol for the search is available from the corresponding author upon request and has not been registered.

### Inclusion and Exclusion Criteria and Search Strategy

Several inclusion and exclusion criteria were specified to narrow the search. For inclusion, studies must have used any *media data* (see the definition in the *Overview* section) as their data source. The outcomes in the study must be related to vaccine sentiment, opinion, uptake, or hesitancy. Although the aim of this study was to look at vaccine hesitancy, this was often done in indirect ways of asking about sentiments regarding vaccines. Uptake can also be another proxy for vaccine acceptance. As social media became a phenomenon in the late 2000s, the search was limited to the year 2010, chosen arbitrarily but corresponding loosely to the year of the H1N1 influenza pandemic in which a vaccine was developed. Imposing a time restriction intentionally did two things: (1) it focused the search on social media platforms (although this is specified in the search terms) and (2) it weighted the search toward capturing more big data methods. Despite the imposed time cutoff and bias toward these methods, non–big data methods for analyzing texts were expected to appear in the search. Regarding exclusion, studies that used social media platforms for recruitment of participants for survey data collection were excluded. Studies using media platforms to conduct natural experiments (eg, introducing social media campaigns) were also excluded. Unpublished manuscripts, protocols, editorials, letters, case reports, commentaries, opinion pieces, narrative reviews, clinical guidelines, and books were also not analyzed.

The search strategy broadly consisted of 2 sets of terms. The first set captured the specified platform of interest to obtain the most popular messaging channels. The second set captured the concept of vaccine hesitancy using synonymic terms. These terms are expressions of the hesitancy concept in a different way. It is important to note that, although these terms are nuanced (eg, antivaccination connotes an absolute rejection of vaccines), they are still part of the overall vaccine hesitancy spectrum. Thus, they were included in their wildcard form. The same search was performed in two different databases: PubMed and Scopus. A summary of the exclusion and inclusion criteria can be found in Textbox 1, and the specific searches can be found in Multimedia Appendix 2.

**Inclusion and exclusion criteria.**

**Inclusion criteria**

- Use of any media data (social media or traditional media) as data source
- Outcome must be related to vaccine sentiment (eg, opinion, uptake, hesitancy, acceptance, or stance)
- Written in English
- Published after 2010

**Exclusion criteria**

- No use of media data as data source
- Use of survey data (asking about social media use as a questionnaire item)
- Use of social media to recruit participants
- Use of social media platform as natural experiment
- Unpublished papers, protocols, editorials, letters, case reports, commentaries, opinion pieces, narratives, clinical guidelines, and books

### Study Selection

Two-step screening was implemented after removing duplicates found in the three databases. Titles and abstracts were screened first as a quick filter for eligibility. Any study not meeting the inclusion criteria (or meeting the exclusion criteria) was removed. Subsequently, the remaining full texts were extracted. Studies that did not meet the eligibility criteria (Textbox 1) during extraction were further removed. All removed studies were classified on their reasons for exclusion. Only JDY screened the articles because of manpower limitations.

### Data Extraction

To reiterate, this review summarized *what* platforms were studied, *how* the data contained were analyzed, and how the studies *built* or *contributed* to the existing work on the media’s influence on vaccine hesitancy. This loosely corresponds to the “concept” portion in the Population-Concept-Context framework of the Joanna Briggs Institute [15]. Accordingly, the four main extracted elements were (1) media platform, (2) analysis method, (3) theories, and (4) findings. Other variables such as (5) the country of focus and (6) language were also included and can be thought to correspond to “context” given the foreseeable diversity in languages and regions of focus. All data were synthesized and charted in Covidence.

### Presentation of Results

The results were separated according to what type of *media* data were used: traditional media or social media. Within each type of media data, a cross-tabulation of the platforms and data analysis methods was presented with accompanying descriptive statistics that illustrated notable trends. As studies can contain one or more platforms or methods, cells in the cross-tabulation are not mutually exclusive and present overlaps. Fully detailed extractions can be found in Multimedia Appendix 3 [17-80] and Multimedia Appendix 4 [81-134]. Trends in any theory were presented descriptively in the text in addition to the countries and languages represented. The *Discussion* section summarizes the major findings and gaps in the literature that uses media data for vaccine hesitancy research and proposes a method moving forward.

## Results

**Figure 1:**
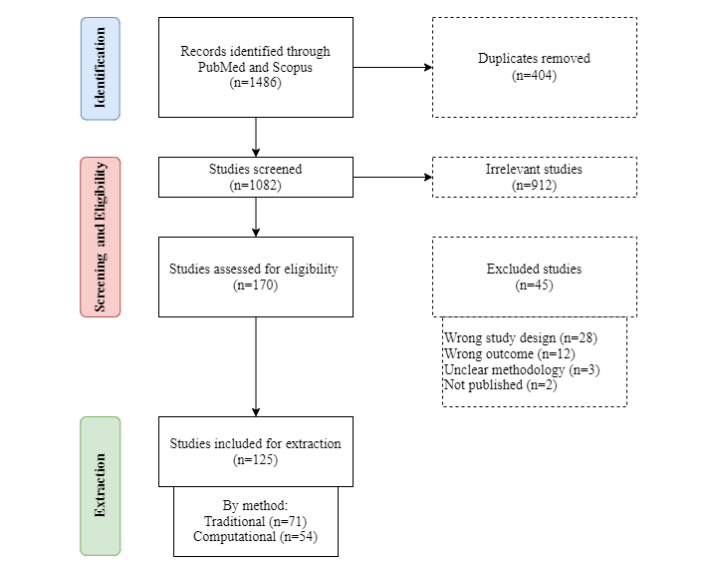
PRISMA-ScR (Preferred Reporting Items for Systematic Reviews and Meta-Analyses extension for Scoping Reviews) flow diagram for this scoping review.

The results of the screening and selection process are presented in the PRISMA-ScR chart (Figure 1). A total of 125 studies were included in this scoping review, of which 71 (56.8%) used traditional methods and 54 (43.2%) used computational methods.

### Traditional Methods

#### Overview

Before the advent of computational big data approaches to analyze media data, several *traditional media data analysis methods* (hereon, *traditional methods* or *noncomputational methods*) were used to research vaccine-related topics. This term distinguishes studies that use media data in a manual way; that is, a way that requires the researcher to individually sort through each data point to extract data. These can be further decomposed into two types: tangential studies and directly related studies.

#### Tangential Studies

Studies with tangential relations include a discussion on vaccines or use of a vaccine-related variable but may not specifically focus on a vaccine outcome as the main variable of interest. There are three subtypes: a focus on a specific population, understanding the nature of information processing, and systematic reviews.

Regarding studies focusing on populations, Leader et al [135] tried to understand the role of “influencers” or “key opinion leaders” on spreading vaccine-related messages in groups of mothers through focus group interviews. They found that influencers posting on vaccine-related issues preferred using information from alternative sources and search engines as opposed to using mainstream information.

Another type of study focused on the nature of information processing in line with the aforementioned category of media studies. An example is the study by Domgaard and Park [136] analyzing how infographic versus SMS text messages may equip users with heightened ability to verify false news in relation to vaccines. Qian et al [137] look at how exposure to negative information may enforce preheld biases and how positive information exposure affects vaccine decision-making. These studies, by focusing more on the psychology of discernment and decision-making, found that the medium (infographics vs text) and connotation (positive or negative) of information transmission are associated with eventual vaccine uptake.

The last type of study was systematic reviews. A Cochrane review looked at the effectiveness of social media in public health interventions [138], with inconclusive findings on overall effectiveness but identifying that studies do not focus on the adverse effects of these interventions. Another systematic review focused on the different methods used for social media monitoring in relation to vaccines [139]. The last review looked specifically at digital interventions with the intention of increasing influenza vaccination among pregnant women [140]. The findings from these 3 studies are largely broad and inconclusive on any effect that public health interventions via social media have on either health outcomes or uptake of vaccination. This can be due to the lack of high-quality, comparable studies that have the same outcome. Notably, two of these systematic reviews consolidated information on experiments, and they were excluded from this review.

Although these studies can be argued to have vaccine-related outcomes as they include vaccine-related data, they are mentioned separately as the primary objectives do not focus on vaccine-related outcomes. Despite their exclusion, these studies highlight the potential of social media–type studies to broaden the scope of research at the public health level, specifically focusing on populations of users, processing of types of information, and public health outcomes from interventions. These factors—populations, processing, and interventions—are all tied closely to the 5 themes identified later.

#### Directly Related Studies

Most studies (65/125, 52%) focused on a direct vaccine outcome and encompassed a variety of countries and languages. The most represented countries were from Europe (France: 3/65, 5%; Italy: 6/65, 9%) as well as the United States (8/65, 12%). Fewer studies came from Asia (3/65, 5%), Africa (3/65, 5%), and the Middle East (1/65, 2%). This diversity in location was also represented in the different languages (where the country or language was not explicitly stated, an inference was made depending on the search terms or the national language of the country): Mandarin Chinese, Cantonese, French, Danish, Italian, Spanish, Hebrew, and English, with the most common being Italian (6/65, 9%) and English because of the multiple English-speaking nations (41/65, 63%). This language diversity will not be reflected in the computational study results, as will be seen.

The platforms and methods used in these studies are summarized in a cross-tabulation (Table 1). Most studies used manual content analysis (43/65, 66%), with a focus on any important themes, topics, frames, or discourse (column 2), and sentiment analysis (21/65, 32%), including any analysis of the tone of vaccine messages, stance on vaccination, polarity in comments, or sentiment classification (column 3) to analyze texts, with few touching on campaign evaluations (5/65, 8%). In the fourth column, some studies track search activity related to vaccines, vaccine coverage, and spread or reach of vaccine-related information (12/65, 18%), highlighting the importance of the SARF framework by Kasperson [10] in vaccine research. The studies included in the table were conducted over a wide assortment of platforms, from traditional media (print media, newspapers, web-based news, and talk shows) to social media (Facebook, Weibo, and Google).

**Table 1:** Traditional analysis methods and media platforms for studies with a direct vaccine-related outcome (N=65)^a,b^.

Media platforms	Analysis methods			
	Content, theme, frame, and discourse analysis	Sentiment, stance, tone, and polarity coding	Activity on the web, media coverage, coverage of vaccines, and misinformation spread	Campaign evaluation
Weibo and Twitter	Becker et al [17] Bonnevie et al [18] Criss et al [19] Griffith et al [20] Hou et al [21] Keim-Malpass et al [22] Marchetti et al [23] Sundstrom et al [24]	Becker et al [17] Criss et al [19] Keim-Malpass et al [22] Marchetti et al [23] Gori et al [25]	Sundstrom et al [24] Aquino et al [26]	Sundstrom et al [24]
YouTube	Marchetti et al [23] Basch et al [27] Fieselmann et al [28] Lahouati et al [29]	Marchetti et al [23] Lahouati et al [29] Covolo et al [30] Donzelli et al [31]	Basch et al [27] Donzelli et al [31]	—^c^
Facebook	Marchetti et al [23] Sundstrom et al [24] Fieselmann et al [28] Bradshaw et al [32] Jamison et al [33] Kalichman et al [34] Orr et al [35] Tustin et al [36] Wawrzuta et al [37] Wiyeh et al [38]	Marchetti et al [23] Tustin et al [36] Loft et al [39] Luisi [40]	Sundstrom et al [24] Aquino et al [26] Loft et al [39] Luisi [40,41]	Sundstrom et al [24] Loft et al [39] Pedersen et al [42]
Websites, mixed media, and blogs	Marchetti et al [23] Orr et al [35] Aechtner [43] Bruel et al [44] Larson et al [45] Moran et al [46] Nugier et al [47] Toth [48] Ward and Budarick [49]	Marchetti et al [23] Larson et al [45] Karapetiantz et al [50] Panatto et al [51] Shoup et al [52]	Suppli et al [53]	—
Q&A^d^ site	Sharon et al [54]	Sharon et al [54]	—	—
Google (search, results, and trends)	Ruiz and Bell [55] Sajjadi et al [56]	—	Aquino et al [26] Suppli et al [53] Diaz et al [57]	—
Pinterest	Guidry et al [58]	—	Guidry et al [58] Mahroum et al [59]	—
Print media, newspapers, and web-based news	Ashwell and Murray [60] Basch et al [61] Casciotti et al [62] Catalan-Matamoros and Elías [63] Colón-López et al [64] Court et al [65] Das et al [66] Kummervold et al [67] Meyer et al [68] Odone et al [69] Olufowote [70] Stephenson et al [71]	Ward and Budarick [49] Casciotti et al [62] Catalan-Matamoros and Elías [63] Das et al [66] Catalan-Matamoros and Peñafiel-Saiz [72]	—	Ward and Budarick [49]
Television talk show	Toth [48]	—	—	—
Documentary	Bradshaw et al [73]	—	—	—
TikTok or Instagram	Fieselmann et al [28] Basch et al [74]	—	Basch et al [74]	—

^a^This table does not include the tangential studies mentioned in the *Tangential Studies* section.

^b^The cells are not mutually exclusive. Studies may appear twice across cells.

^c^Not available. No studies exist using this media platform and analysis method.

^d^Q&A: question and answer.

A minority explicitly stated a theoretical framework that drives the analysis. Ward and Budarick [49] used a discursive legitimization strategy and ideological square theories to evaluate the use of anecdote and emotionality by The Daily Telegraph to push provaccine messaging in a campaign to increase vaccination. A study focusing on discourse used repertoire analysis to understand how parents’ repertoires in distrust contribute to a delegitimization of systems propping up medical services, research, and government authorities [48]. Another study on repertoire echoes those using framing theories to understand how positive or negative framing could coerce behavior [60]. In total, 3% (2/65) of the studies looked at the influence of persuasion as a tactic in the delivery of text [46] and as a guide to framing certain cues to influence vaccine uptake behavior [43]. These studies used persuasion theory and the ELM of persuasion to guide discussion. Persuasion theory also connects to other influence theories such as social influence theory, in which individuals change their behaviors to meet the demands of a social environment. In total, 2% (1/65) of the studies analyzed how mothers changed their behaviors within Facebook networks around antivaccination advocates [32]. A total of 3% (2/65) of the studies, conducted by Luisi [40,41], directly used the SARF and the HBM to operationalize concepts within each framework, using human papillomavirus (HPV) vaccination discussions on Facebook as data.

Studies using content and discourse analysis have strong theoretical roots in the social sciences. However, few studies in which these methods were used to study vaccines explicitly mentioned a theory driving their study (12/65, 18%). If manual analyses, which are limited to the physical capacity of data processing, are already theoretically shaky, we expect an even weaker theoretical focus using computational methods.

### Computational Methods

A total of 43.2% (54/125) of the studies used computational (big data) methods. There were obvious trends in language, region, and which vaccines were studied. Most of the studies (36/54, 67%) used English-language media data, with a small representation from other European languages (Italian: 5/54, 9%; Dutch: 1/54, 2%; Polish: 1/54, 2%; French: 2/54, 4%), which are often studied alongside English in the same study. Italian is an exception as it is studied independently of English compared with the other European languages. Several East Asian languages were represented as well, with simplified Mandarin Chinese (5/54, 9%), Korean (2/54, 4%), and Japanese (1/54, 2%). In total, 2% (1/54) of the studies used multiple languages from various contents to do a comparison by region as well [81].

In media data analysis, the geographical location or region of study (and, thereby, the population) is not often explicitly stated and, even when done so, it can be ambiguous. Most often, “geography” is determined by explicit mention of a region of interest or inference through pulling of data with a geographical focus (eg, pulling tweets from geotagged posts from the United States) or a language focus (eg, parsing data from a platform published mostly in Japanese). As a result, language often correlates with region, but this is not always the case, especially for a *lingua franca* such as English, which disallows mapping one-to-one because of the many countries that speak it. Despite this deductive approach, 26% (14/54) of the studies did not specify any location but contained English-language media data. Most studies were conducted with the United States as a geographic region of interest (17/54, 31%), followed by China (5/54, 9%) and Italy (5/54, 9%). In total, 9% (5/54) of the studies took a comparative approach and contained multiple jurisdictions of comparative interest, even including 20% (1/5) that adopted a global comparative approach [82]. Compared with studies using traditional methods, we observed limited representativeness of countries and languages. This was due in part to the necessity of parsing and understanding a large quantity of language and the limited language processing tools developed for smaller languages. For countries that are primarily English-speaking—or English-expressing, for capturing web-based information—but not represented here, there are likely to be more studies in these regions as language processing tools are popularized in public health.

Regarding the types of vaccines studied, it is important to note that time censoring of the review would bias the data set to more recent vaccine issues. Most studies (20/54, 37%) focused on the COVID-19 vaccine and were published within the last 2 years. The other popular category of vaccines was not any specific vaccine but, rather, vaccines in general (17/54, 31%), focusing on the overall sentiment and topics related to vaccination. A smaller minority focused on HPV (4/54, 7%); influenza (3/54, 6%); childhood vaccinations (3/54, 6%); maternal vaccinations (2/54, 4%); and the measles, mumps, and rubella vaccine (1/54, 2%).

All the studies included in this section (54/54, 100%) were published in or after 2016. Among them, a diverse selection of platforms and analysis methods were used. Table 2 cross-tabulates these 2 variables in a similar fashion to Table 1, revealing some trends. Overwhelmingly, Twitter was the most popular platform, with 57% (31/54) of studies using it. It is also more represented across the different analysis methods relative to other platforms. This is different when compared with the traditional methods table, where Twitter studies were uncommon. This trend was the opposite for print and news media and web-based news, with less representation as a platform when computational methods were used. The other platforms were novel in Table 2. For example, different search engines appeared: Baidu (China) and Naver (Korea). Parler, a microblogging platform, was also novel.

What types of analysis methods were used? The methods were categorized into the following eight broadly non–mutually exclusive groups: (1) sentiment analysis, (2) topic modeling, (3) semantic network analysis, (4) projections, (5) feature extraction, (6) image analysis, (7) descriptive studies, and (8) machine classification. Sentiment analysis studies (31/54, 57%) assessed various issues, such as stance [81,85,86], emotions [89,117], and polarity [91,123], and the following algorithms, which were used to determine the aforementioned issues, were diverse: Bidirectional Encoder Representations from Transformers, classification tree, K-nearest neighbors, multinomial naïve Bayes, random forest, robust optimized Bidirectional Encoder Representations from Transformers pretraining approach, support vector machine, and Valence Aware Dictionary and Sentiment Reasoner. Topic modeling (18/54, 33%) was a close second in popularity and focused on distilling latent topics within a corpus. The most common method for topic modeling was latent Dirichlet allocation coupled with other methods to look at topic clustering (related to semantic network analysis) or at inter- and intratopic distinctiveness [97]. The studies focused on sentiment analysis and topic modeling were, in part, a continued momentum of traditional research methods that focused on distilling these aspects from the text.

Semantic network analysis (17/54, 31%) focused on understanding the interaction and transfer of information and ideas within specific networks. Methods ranged from cluster analysis using Gelphi [103,106], latent space modeling [100], exponential random graph modeling [126], and the Louvain algorithm for community detection [102,105,106,126]. The remaining analysis types were represented in smaller numbers. A total of 2% (1/54) of the studies used a behavioral dynamics model—inspired by epidemiological models on susceptibility and infected and resistant states of being—to analyze opinion transmission models [116]. Another 4% (2/54) of projection studies used media data and regression models to predict vaccination rates and epidemic size [108,120]. Feature extraction was only found in 2% (1/54) of the studies, in which Lyu et al [109] extracted variables such as demographics, social capital, income, and political affiliation from a corpus of tweets and associated these features with vaccine stance using logistic regression. Image analysis, also known as *computer vision,* was represented in 2% (1/54) of the studies, in which Wang et al [132] used a multimodal network analysis to detect antivaccine messages on Instagram.

In total, 2 methods were included as separate groups despite their overlap with other methods. For example, all the studies likely contained a descriptive portion in their results. As such, descriptive studies were those that were only descriptive of their categories of interest, sentiment analysis aside. Examples include those describing group counts, changes over time, or other unique ways of data visualization [81,110,111,118,121,130]. Similarly, sentiment analysis studies sometimes include the development of a supervised machine learning model. Thus, the machine classifier method only contained 2% (1/54) of the studies that focused exclusively on machine classifying, which detailed the development of a classification model that identifies false HPV information [112].

Although diverse, computational studies also share a unifying theme with traditional method studies, which is the deficiency of the theoretical focus driving these studies. Even fewer studies using computational methods had a theoretical basis (6/54, 11%). Of the 6 studies that did, only 1 (17%) focused on a health behavior model [110], and the others used more generalized theories [81,97,108] and marketing [121].

**Table 2:** Computational analysis methods and media platforms (N=54).

Media platform	Analysis methods							
	Sentiment analysis	Topic modeling	Semantic network analysis	Projection	Feature extraction	Image analysis	Description^a^	Machine classifier^b^
Twitter	Martin et al [81] Liew and Lee [82] Ajovalasit et al [83] Blankenship et al [84] Cotfas et al [85] Deiner et al [86] Du et al [87] Gesualdo et al [88] Hu et al [89] Lyu et al [90] Monselise et al [91] Piedrahita-Valdés et al [92] Shim et al [93] Tavoschi et al [94] Yan et al [95] Yousefinaghani et al [96]	Blankenship et al [84] Cotfas et al [85] Hu et al [89] Lyu et al [90] Monselise et al [91] Shim et al [93] Yan et al [95] Argyris et al [97] Dunn et al [98] Guntuku et al [99] Jiang et al [100]	Martin et al [81] Jiang et al [100] Benis et al [101] Boucher et al [102] Featherston et al [103] Germani et al [104] Gunaratne et al [105] Lutkenhaus et al [106] Marcec and Likic [107]	Pananos et al [108]	Lyu et al [109]	—^c^	Martin et al [81] Guidry et al [110] Kummervold et al [111]	Ajovalasit et al [83] Du et al [87] Argyris et al [97] Dunn et al [98] Tomaszewski et al [112]
Weibo	Chen et al [113] Zhang et al [114]	Hu et al [115]	—	Yin et al [116]	—	—	—	—
Facebook	Deiner et al [86] Klimiuk et al [117] Schmidt et al [118] Zhang et al [119]	Schmidt et al [118] Zhang et al [119]	Bar-Lev et al [120]	Bar-Lev et al [120]	—	—	Furini [121]	—
Parler	—	Baines et al [122]	—	—	—	—	—	—
Forum, blog, or Reddit	Martin et al [81] Yan et al [95] Melton et al [123]	Yan et al [95]	Martin et al [81]	—	—	—	Martin et al [81]	—
Websites (assorted)	Kang et al [124]	Okuhara et al [125]	Bar-Lev et al [120] Kang et al [124] Cafiero et al [126]	Bar-Lev et al [120]	—	—	—	—
Web-based news or media cloud	Getman et al [127]	—	Getman et al [127]	—	—	—	—	—
Baidu, Google, or Naver	Chen et al [113] Lee et al [128] Porreca et al [129]	—	Porreca et al [129]	—	—	—	—	—
Multiple	Powell et al [130]	DeDominicis et al [131]	DeDominicis et al [131]	—	—	—	Powell et al [130]	—
Instagram	Lee et al [128]	—	—	—	—	Wang et al [132]	—	—
Q&A^d^ site	Luo et al [133]	Luo et al [133]	—	—	—	—	—	—
YouTube	Porreca et al [129]	—	Porreca et al [129]	—	—	—	—	—

^a^Nearly all studies included descriptive statistics of their data set and outcome of interest. The descriptive classification included studies that were descriptive of categories other than sentiment (eg, categories of vaccine confidence or vaccine stance).

^b^Machine classifier studies only developed a classifier without further analysis. Often, but not always, studies using sentiment analysis or topic modeling also used machine classifiers; however, this table does not distinguish this.

^c^Not available. No studies exist using this social media platform and analysis method.

^d^Q&A: question and answer.

### Themes

Although the studies were diverse in methods and their outcomes of focus, five themes were distilled that summarize this diversity: antivaccination themes, provaccination themes, framing, coverage and activity, and response of activity to certain events.

A set of studies (39/125, 31.2%) focused on what antivaccination topics arose. The most commonly recurring theme was a distrust of government institutions [18,20,21,36,37,43,70,75,76,81, 102,124,131] or health institutions [32,35,36,49,67,93,100,125] or the idea of pharmaceutical companies profiteering off individuals [20,29,122,125]. This spilled into a related conversation about the infringement of civil liberties when individuals feel they are forced or mandated to receive a vaccine [37,46,47,64,73,131]. Often, the narratives can also be full of misinformation [27,73] or conspiracy theories [35,37,58,70,100,117], both featuring heavily when in antivaccination messages and accompanied by anger, fear, or frustration [96]. These sentiments were also paralleled by a general concern about specific vaccines themselves, especially in relation to their overall perceived safety or efficacy (including side effects) [35,38,44,45,51,75,81,93,102,117,122,123,125, 128], their constitution or ingredients [18], the adverse events around them [18,19,27,61,66], and their unnaturalness [32,47].

By contrast, other studies (8/125, 6.4%) focused on provaccination topics that emerged (although in fewer studies, understandably so as vaccine hesitancy was the focus). The most common theme was the use of scientific research and a constant reinforcement of vaccine safety and efficacy [19,62,77,100,125]. The second most common theme was how having an empathetic connection may lead to perception of vaccines in a more positive light. A total of 0.8% (1/125) of the studies found that knowing an afflicted person with HPV appeared in provaccination messages [38]. Another study (1/125, 0.8%) found that, for childhood vaccinations, vaccine advocates focused on the impact of vaccine hesitancy on children to encourage others to vaccinate their children [131].

The existence of antivaccination and provaccination topics alludes to the importance of who is delivering a message and how it is delivered [54]—captured in the third theme, framing. Regarding who delivers the message, general practitioners are used so that transmitted messages are more reliable [44,54,129] and engaged with [133], and sources from governments or professional associations are most used for credibility or transparency [56,63]. The opposite is true, where negative information is usually associated with less professional institutions [51,52]. This may be especially important in a landscape in which posts or content are likely generated by lay consumers or users [22,125,133]. The use of parents and mothers as messengers elicits a better generation of concern [49]. In addition, writers and journalists influenced by both provaccination and antivaccination camps are shown to continually reignite the debate on vaccination [106]. Regarding how the message is conveyed, personal stories, which are shown to be more engaging [39], are a tool used by both sides to enforce their viewpoints as correct [24] (such as the use of anecdotes on antivaccination websites [46] or the use of personal stories to encourage positive vaccination dialogue [39,42]). Another tactic used for framing, especially from the antivaccination side, is the use of shocking images or appeals to emotion through testimony to convince others of the antivaccination agenda [47,104]. Often, these antivaccination messages misuse scientific evidence [121,127] and loss-framed messaging [112] to transmit their ideas. These tactics may allude to a more generalized use of risk-amplifying messages to elicit reactions [41]. Framing also inadvertently occurs when using certain terms. In total, 0.8% (1/125) of the studies looked at how antivaccination characterizes vaccine-hesitant groups as ignorant, deviant, lacking access to vaccination (as opposed to being unwilling), pitied, and needing help [65]. In summary, who delivers the message, their background, and how they say it are all important in vaccine hesitancy research.

Closely related to framing is the relative amount of coverage, activity, or engagement on the web of provaccination and antivaccination communities. Most studies in this theme (12/125, 9.6%) found that any negative or antivaccination coverage or messages were generally more prevalent and engaged with (shared, viewed, retweeted, and liked) [21,25,29-31,33,50,55,68,71,78,84]. There were 1.6% (2/125) of studies in the opposite direction, finding that positive vaccination messages received more engagement [58,96] despite the existence of a higher quantity of antivaccination videos. Some studies (8/125, 6.4%) went further to establish an association between coverage—both the type and amount of coverage—and vaccine uptake. In total, 0.8% (1/125) of the studies found that a higher number of tweets, Facebook posts, and internet searches in an area were associated with lower measles, mumps, and rubella vaccine coverage [26]. This was corroborated by 1.6% (2/125) of the studies: an infodemic study that found an association between higher social media traffic and higher hesitancy [120] and a study that found that more exposure to HPV-related tweets explained variance in coverage [134]. Another study (1/125, 0.8%) found that more negative coverage meant less uptake of childhood vaccination [72]. This was corroborated by 1.6% (2/125) of the studies—a study looking at how adverse event reporting meant less vaccination [53] and a study that showed that discourse on HPV vaccines focusing on negative tones was correlated with more barriers to HPV vaccination [40]. However, the opposite was found in a Chinese study, which noted that increasing vaccine-related discussions correlated with an increasing number of vaccinated individuals [95]. Another study (1/125, 0.8%) found that more tailored messages to specific communities would lead to higher proactiveness in certain parts of the population to get vaccinated [101]. Another set of studies (9/125, 7.2%) looked at how vaccine-discussing communities engaged with each other. An example of this is the finding that antivaccination groups discussed vaccination issues much earlier [34]; are deeply fragmented in their beliefs, which spiral into radical communities [126]; and are part of a larger robust network of vaccine-hesitant individuals [97,98,127]. This robustness is also found in provaccination networks [124]. Overall, vaccines are a very polarizing topic, partly because of the ideological isolation and minimal interaction between provaccination and antivaccination groups [105], as well as other minority groups [99], and the existence of echo chambers that arise because of selective consumption of vaccine information [118].

The last theme captures how discussion of vaccines clusters around events, indicating a reactive public over time [64,87,89-91,94,107]. Overall, the conversation around vaccines usually follows certain occurrences or events in what is termed as crisis phases by Furini [121]. Diaz et al [57] found that there was increased search activity regarding vaccines and infertility following the US Center for Disease Control and Prevention emergency approval of COVID-19 vaccines. Interactions on Twitter increased in response to political events, suggesting disorientation [83,85]. Mahroum et al [59] found that, in an influenza vaccine scandal (the Fluad case), regions affected by the scandal had more related web search activity, suggesting a localized search behavior. Odone et al [69] corroborate this by highlighting that reports of deaths were the main signal that prompted more searches on the topic. A similar finding was also noted by Deiner et al [86], who showed that provaccination posts were correlated with a reporting of US cases (with antivaccination posts constantly happening in the background). There is also a focus on the associations of this increased activity. Chen et al [113] looked at how the vaccine crisis of the Kangtai hepatitis B virus raised public attention and negative sentiments on the web in China. Dunn et al [134] found that exposure to more HPV-related tweets explained a variance in coverage of the HPV vaccine. Adverse event reporting also produces a more emotional response that leads to a decline in positive sentiments about vaccines [114]. Another set of studies (2/125, 1.6%) looked at the content of the messages, which overlaps with the aforementioned negative topic theme. In total, 0.8% (1/125) of the studies looked at how, during the peak season for influenza, more conspiracy theories about vaccination would occur [110]. Another study (1/125, 0.8%) looked at how the public had episodic expressions of distrust toward the Chinese government immediately after a vaccine-related scandal [115]. Although this theme discusses how the public reacts, there is also considerable overlap with the other themes in terms of what is being said as a reaction.

## Discussion

### Principal Findings

This review consolidated the current literature on the use of media data—both traditional and social media—to study vaccine hesitancy. This was done through three objectives: (1) summarizing media platforms; (2) summarizing analysis methods; and (3) understanding how the included studies build or contribute to the body of knowledge of the media’s influence on vaccine hesitancy and, thereby, on public health. In doing so, this study aimed to bridge the fields of health behavior, computer science, and public health. A total of 125 studies were included, of which 71 (56.8%) used traditional research methods and 54 (43.2%) used big data (computational methods). The studies focused on the following five themes: identifying antivaccination topics; identifying provaccination topics; framing (who says what and how); the coverage, activity, and engagement in provaccination and antivaccination communities; and how the public reacts to events.

Overall, there is plurality in the analytical methods used. Several methods prevailed. For the traditional methods, most studies (43/71, 61%) focused on using content analysis, thematic analysis, or framing analysis, with other methods such as sentiment, stance, tone, or polarity coding also being popular. This preference was extended, perhaps naturally because of momentum in the field of vaccine hesitancy to focus on sentiment and topics, to studies using computational methods. Studies using network analysis and feature extraction were present but fewer (16/54, 30%). This could be due to a time lag in the arrival of big data analysis tools for academic research in this direction. Interestingly, all studies using computational methods (54/125, 43.2%) were published in or after 2016, indicating a relatively recent interest in this area. In the coming years, there may be growth in the computational field, especially regarding more advanced network analyses and feature extraction. This growth offers new insights to researchers, enabling them to reach new conclusions and challenge existing theories, thereby revolutionizing the way vaccine hesitancy studies are conducted.

However, this revolution is not only due to advances in computing. In parallel, the creation of new platforms will also shape the ways in which users engage with information. The different platforms used in the included studies span blogging sites, microblogging sites, newspapers, image-based social media platforms (Instagram), video-based social media platforms (YouTube), search engines, and question-answering sites. The growth of live streaming on platforms such as Instagram reels, TikTok, Bilibili (Chinese video streaming platform), and Twitch is likely to pivot analysis methods in the direction of *computer vision*, and preferences for more advanced methods may follow suit. In this review, this shift was observed. The studies using manual methods (71/125, 56.8%) focused more on traditional media, whereas those using computational methods targeted social media and microblogging platforms. Thus, the diversification of platforms parallels the advances in methods. Together, their parallel growth synergistically shapes the epistemological paradigms of media use in vaccine hesitancy research.

Despite fervor on the growth of this field, a glaring shortcoming misroutes it—a lack of theoretical foundation. Missing a theoretical focus portends the use of methods only for the sake of novelty and not necessarily informativeness. A corroborating finding speaking to this point is some studies’ justification of publication on the grounds of a novel approach to data analysis when the analysis only applied methods to a different data source or platform. Another corroboration is the inadvertent lack of computational methods used to analyze traditional media, possibly because of the attractiveness of big data methods (ie, preferences to analyze social media because of novelty). Although this contributes to an overall body of knowledge in vaccine hesitancy research, it disorganizes the trajectory of the field as findings are not built on the cornerstones already set by theories in health behavior, vaccine hesitancy, and public health. Thus, it makes it difficult to draw any conclusive findings on the media’s real influence on vaccine hesitancy as measured variables and outcomes differ. Using a theory-driven approach can counter this trend, making the consolidation of findings more cogent. By anchoring these studies on health behavior or information proliferation theories, the parallel development of media data and public health research can be bridged while simultaneously addressing the blind spot of theoretical weakness.

Few studies in this review (19/125, 15.2%) exhibited a theory-driven approach. Bradshaw et al [73] used social influence theory to guide the discussion on how antivaccination advocates on Facebook inadvertently used informational and normative influence processes to shape first-time mothers’ vaccination sentiments. The discussion extended to how the Facebook network, being geographically unrestricted, may promote vaccine refusal in line with digital identity formation, expanding the realm of influence on vaccination. Aechtner [43] focused on persuasion cues derived from the ELM for persuasion to label and guide discussion of an Australian countervaccine lobby group. In total, 1.6% (2/125) of the studies, conducted by Luisi [40,41], also used two of the most prominent theories in health behavior and psychology to guide coding. Of these 2 studies, 1 (50%) looked at the amplification potential of messages by measuring the concepts of the SARF on Facebook posts [41]. The other study used a similar method but used the HBM to guide the labeling of the concepts present in the messages on Facebook [40]. Pananos et al [108] took an entirely different approach, not using a health behavior model but rather one from mathematics. In their study, they used the theory of critical transitions and Twitter data to predict how critical periods in the vaccination course (around a “tipping point”) may affect the course of epidemics. If a study did not explicitly invoke a theory, it could arrive at one or more conclusions that were captured in one or more theoretical concepts. One example of this is the study finding that there are emotion-based risk expressions in antivaccination groups (risk as an emotion concept) [96]. These studies are only a sample of what can be done with theoretical guidance.

There are 2 additional implications of a theory-based approach. The first is that these novel methods in media analysis are unlikely to replace existing methods in vaccine hesitancy research; rather, they are an extension and complement to them. Survey methods have been validated in public health for the past 50 years in its research, and guided questions have been drafted to draw conclusions on the complex relationship among factors that drive behaviors. As such, there are some conclusions drawn from survey data that are difficult to obtain using media data. An example is the causative analysis of vaccine perceptions and uptake. Media data are just 1 factor in a complex information network, and there are confounding issues (demographics, preconceived beliefs, and heuristics, to name a few) in drawing causative conclusions on individual or population vaccine hesitancy because of exposure to information. From this review, it is apparent that there is a paucity of studies exploring associative links between a specific media channel and vaccine uptake. Thus, media data analyses will likely only complement the existing public health research paradigm until more advances are made. The second implication is that refocusing on theory (a defocusing on methods) allows for a better identification of gaps in the literature. Researchers are better able to identify which platforms, concepts, or relationships need stronger testing and empirical support if structured by a framework. These 2 implications delineate the scope of what media studies in vaccine research accomplish in terms of pushing forward the vaccine hesitancy research agenda.

### Future Directions

On the basis of these themes, there are several open research areas for further exploration. The first is to understand how trust and distrust toward institutions (government and health care) may influence vaccination. A common theme of antivaccination worldwide appears to be rooted in distrust and suspicion—which translates to fear or disobedience—on the part of the public. This may translate to conspiracy theories and misinformation within antivaccination communities. Although media data can aid in the identification and classification of topics and understanding how they spread in networks, there is pending work on understanding the association between trust and adherence to public health measures. Second, and closely related, is research on understanding how misinformation spreads. This field of work will likely involve health psychologists, computer scientists, public health experts, and media researchers as it involves understanding how information signals are generated, spread, and processed; what signals are important in shaping risk perception; and how the timing of this matters. This field of misinformation and understanding how to combat it, with the implications for public health, will be a huge challenge in the era of big data and public health. The last area is the effective communication of governments and the pharmaceutical industry in addressing any vaccine concerns, from constitution and side effects to any other vaccine-related events. Evaluations of governments on vaccine communication should be performed and benchmarked against WHO-prescribed standards such as those laid out in the COVID-19 Vaccine Safety Manual [141] or the Managing Vaccine-Related Events guide [142], with the aim of identifying successful case studies on vaccine communications. These are several areas of suggested research on vaccine hesitancy moving forward.

### Limitations

There are several limitations to this study. First, data were extracted by only 1 reviewer. This affects the inclusion criteria and extraction process through a combination of selection bias and manpower limitations. A predefined standardized extraction form partially diminished any biases in data extraction. In addition, as the review only consolidates and describes platforms, methods, and contributions to the field of study without concluding about results or effect size, the introduced bias has a marginal influence on the findings.

The second limitation is the left censorship of year in the search criteria. By including studies only conducted after 2010, there is a stronger representation of studies that used social media platforms and computational methods. As vaccine hesitancy and traditional media analysis are not new issues (ie, they were present before social media), there are relevant studies that have not been included. However, this is intentional. An objective was to have a closer look at the diversity in platforms and methods in recent years. Imposing a time restriction homed in on reaching this objective. Regarding concerns about the representativeness of the included studies, there already was an emerging trend in preference for platform and analysis method without the necessity to include every study (ie, a saturation in data findings). This saturation also diminished the biases of only having 1 reviewer.

However, this saturation in data does not preclude that rapid changes in the field will produce new uses of platforms and analyses methods, especially as new developments happen in the fields of computer science and natural language processing. The third limitation extending from the second is the inclusion of studies only in English. There is evidence in the review that analyses in other widely spoken languages such as Chinese and French are emerging. The language of publication is important as the foundation of media studies and natural *language* processing tries to parse meaning from language, with different languages analyzed through a different set of linguistic tools. These different tools, coupled with an inherent difference in language structure, may reveal alternative approaches to distill meaning and connotations from words. Furthermore, studies using non-English languages to analyze vaccine hesitancy could also have implications for global health as many of these non-English languages are spoken by a large portion of the global population (eg, Chinese and Spanish). For these reasons, excluding non-English papers biases the comprehensiveness of the methods and platforms presented.

The last major limitation is the exclusion of studies that used surveys or cross-sectional data. This was explicitly included in the search terms to exclude studies that used surveys to ask about the use of media or the effects of media and to focus the body of studies on those that only used media studies as the main source of data. Although successful, this search excluded studies that used both survey and media data to study vaccine hesitancy. Thus, this major limitation restricts the comprehensiveness of the included studies, and a separate scoping review assessing the dual use of traditional tools and media data is required.

### Conclusions

Our findings illustrate a variegation of media platforms and analysis methods for vaccine hesitancy research as well as 5 themes of focus. The first was the focus of antivaccination themes on the distrust of institutions, violations of civil liberties, the spread of misinformation and conspiracy theories, and concerns about specific vaccines. The second was the focus of provaccination themes on the use of scientific literature to support vaccine safety. The third was the importance of who delivers the message and how the way it is framed shapes the reception of vaccine opinion. The fourth was that coverage mostly centers on negative content and also circulates within echo chambers in both vaccination camps, indicating deeply fractured communities. The last theme was that the public responds to focusing events, suggesting volatile periods in which misinformation and conspiracy information can circulate. Despite the diversity in study types and platforms, these findings are consistent across both traditional and computational methods.

This burgeoning field—known as *digital epidemiology* or *infodemiology*—will continue diversifying as new media platforms arise and more tools from computer science trickle and become commonplace in public health research. This heterogeneity, although inspiring for new avenues of research, should also be met with cautious excitement. Researchers inclined to join this field should fully understand that media data analysis methods are meant to supplement—not supplant—current practices in public health research. A way to ensure this understanding is to establish a theoretical focus of the research before method or platform selection. In doing so, the mentality of adopting trending methods is avoided, there is a systematic consolidation in the synthesis of findings, and a coherent paradigm in the subfield of media data research on vaccine hesitancy can be established.

## Appendix

Multimedia Appendix 1 PRISMA-ScR (Preferred Reporting Items for Systematic Reviews and Meta-Analyses extension for Scoping Reviews) checklist.

Multimedia Appendix 2 Search terms and dates.

Multimedia Appendix 3 Detailed data extraction for traditional method studies.

Multimedia Appendix 4 Detailed data extraction for computational method studies.

## Abbreviations

ELM: elaboration likelihood model
HBM: health belief model
HPV: human papillomavirus
PRISMA-ScR: Preferred Reporting Items for Systematic Reviews and Meta-Analyses extension for Scoping Reviews
SARF: social amplification of risk framework
WHO: World Health Organization
