# Supplementary material for: Media Data and Vaccine Hesitancy: Scoping Review
Source: JMIR Infodemiology. 2022 Aug 10;2(2):e37300. doi: 10.2196/37300 (PMC9987198; doi:10.2196/37300)
Supplement: Multimedia Appendix 2 [file infodemiology_v2i2e37300_app2.pdf]

PubMed:

Search conducted on December 12, 2021:

((((media OR newspaper* OR television OR radio OR magazine OR "social media" OR twitter OR tweets OR reddit OR facebook OR weibo OR forum OR whatsapp OR telegram OR wechat OR weixin OR snapchat OR qq OR kik OR kakao* OR LINE OR youtube OR internet))AND (vaccin* OR anti-vax OR anti-vaccin* OR pro-vax OR pro-vaccin*)) AND (hesitan* OR hesitat* OR confiden* OR refus*)) NOT (survey) NOT (cross-sectional) Filters: from 2010/1/1 - 3000/12/12

Additional search added for Instagram and TikTok into search terms, done on June 10 2022 (same censor date)

((((Instagram OR tiktok))AND (vaccin* OR anti-vax OR anti-vaccin* OR pro-vax OR pro-vaccin*)) AND (hesitan* OR hesitat* OR confiden* OR refus*)) NOT (survey) NOT (cross-sectional) Filters: from 2010/1/1 - 2021/12/12

Scopus:

Search conducted on December 12, 2021:

(TITLE-ABS-KEY (media OR newspaper* OR television OR radio OR magazine OR "social media" OR twitter OR tweets OR reddit OR facebook OR weibo OR forum OR whatsapp OR telegram OR wechat OR weixin OR snapchat OR qq OR kik OR kakao* OR line OR youtube OR internet) AND TITLE-ABS-KEY (vaccin* OR anti-vax OR anti-vaccine OR pro-vax OR pro-vaccine) AND TITLE-ABS-KEY (hesitan* OR hesitat* OR confiden* OR refus*) AND NOT TITLE-ABS-KEY (survey) AND NOT TITLE-ABS-KEY (cross-sectional)) AND (LIMIT-TO (DOCTYPE , "ar")) AND (LIMIT-TO (LANGUAGE , "English")) AND (LIMIT-TO (SRCTYPE , "j")) AND (LIMIT-TO (PUBYEAR , 2021) OR LIMIT-TO (PUBYEAR , 2020) OR LIMIT-TO (PUBYEAR , 2019) OR LIMIT-TO (PUBYEAR , 2018) OR LIMIT-TO (PUBYEAR , 2017) OR LIMIT-TO (PUBYEAR , 2016) OR LIMIT-TO (PUBYEAR , 2015) OR LIMIT-TO (PUBYEAR , 2014) OR LIMIT-TO (PUBYEAR , 2013) OR LIMIT-TO (PUBYEAR , 2012) OR LIMIT-TO (PUBYEAR , 2011) OR LIMIT-TO (PUBYEAR , 2010))

Additional search added for Instagram and TikTok into search terms, done on June 10 2022 (same censor date)

(TITLE-ABS-KEY (*instagram* OR *tiktok*) AND TITLE-ABS-KEY (*vaccin** OR *anti-vax* OR *anti-vaccine* OR *pro-vax* OR *pro-vaccine*) AND TITLE-ABS-KEY (*hesitan** OR *hesitat** OR *confiden** OR *refus**) AND NOT TITLE-ABS-KEY (*survey*) AND NOT TITLE-ABS-KEY (*cross-sectional*)) AND PUBYEAR > *2009* AND PUBYEAR < *2022* AND PUBYEAR > *2009* AND PUBYEAR < *2022*
