# Supplementary material for: Media Data and Vaccine Hesitancy: Scoping Review
Source: JMIR Infodemiology. 2022 Aug 10;2(2):e37300. doi: 10.2196/37300 (PMC9987198; doi:10.2196/37300)
Supplement: Multimedia Appendix 3 [file infodemiology_v2i2e37300_app3.pdf]

Author(s)	Ref. No.	Vaccine(s)	Language(s)	Theories	Platform(s)	Main method(s)	Findings
Aechtner T	[49]	General	English	Elaboration likelihood model of persuasion	Lobby group blog	Thematic coding	Themes: Big Pharma and governments are censoring data for financial gain, pro-vaccine sentiments counter civil liberties, vaccine risks (toxicity of ingredients).
Aquino F; Donzelli G; De Franco E; Privitera G; Lopalco PL; Carducci A	[32]	MMR	Italian		Online search trends<br>Twitter<br>Facebook	Online engagement	More Tweets, Facebook posts, and internet search activity associated with lower MMR coverage.
Ashwell D; Murray N	[66]	General	English	Agenda setting, framing theory	Australia and New Zealand newspapers	Content analysis<br>Frame analysis	News media messages were predominantly positively framed in Australia and New Zealand.<br><br>Positive vaccination reporting may engender resistance to vaccination among vaccine-hesitant
Basch CH; Kecojevic A; Wagner VH	[67]	COVID-19	English		U.S. Newspapers	Thematic coding	Seven themes: Johnson and Johnson vaccine characteristics, describing adverse events, emphasis of rare event and vaccine is safe, health authorities’ investigation, pause in administration of Johnson and Johnson vaccine, vaccine fear and anxiety in increased hesitancy, and Johnson and Johnson’s role and impact in vaccine development.
Basch CH; Meleo-Erwin Z; Fera J; Jaime C; Basch CE	[80]	COVID-19	English		TikTok	Content analysis<br>Online engagement	Discouraging vaccination videos showed a parody of adverse reaction<br><br>Several misinformative videos: indicating vaccine available even though not available
Basch CH; Zybert P; Reeves R; Basch CE	[33]	Childhood	English		YouTube	Content analysis<br>Online engagement	Sources of videos: consumers, TV-based or internet-based news, and individual health professionals<br><br>Topics covered: autism causality, undisclosed or poorly understood risks, adverse reactions, thimerosol or mercury in vaccines.

Author(s)	Ref. No.	Vaccine(s)	Language(s)	Theories	Platform(s)	Main method(s)	Findings
							Most videos discouraged use of vaccines
Becker BF; Larson HJ; Bonhoeffer J; van Mulligen EM; Kors JA; Sturkenboom MC	[23]	Pediatric pentavalent vaccine	English		Twitter	Content analysis<br>Tone analysis	Few interactions of Tweets about pediatric pentavalent vaccine<br><br>Tweets contain information on other websites and information links<br><br>Majority of Tweets were positive, corresponding to country-level events
Bonnevie, E.; Gallegos-Jeffrey, A.; Goldbarg, J.; Byrd, B.; Smyser, J.	[24]	COVID-19	English		Twitter	Thematic coding	Anti-vaccination conversation themes: mistrust in federal health authorities, suspicion about vaccine ingredients, and doubts about trial.
Bradshaw AS; Shelton SS; Wollney E; Treise D; Auguste K	[38]	HPV	English	Social Influence Theory	Facebook group	Thematic coding	Anti-vaccination advocates used informational and normative influence processes.<br><br>Themes of anti-vaccination advocates: advocate natural solutions, maternal empowerment, distrust of conventional medicine establishment, fear appeals, “Russian Roulette” risk benefit analysis, and misinformation spreading
Bradshaw AS; Treise D; Shelton SS; Cretul M; Raisa A; Bajalia A; Peek D	[79]	HPV	English	Grounded theory	Documentary series	Thematic coding	Themes: perceived solidified science, collusion and conspiracy, canary in the coal mine, fear appeals, and morality and necessity of individual choice.
Bruel S; Peyrard-	[50]	HPV	French		Web forum	Thematic coding	Use of general practitioner as a reference to develop a reliable discourse.

Author(s)	Ref. No.	Vaccine(s)	Language(s)	Theories	Platform(s)	Main method(s)	Findings
Chevrier K; Ginzarly M; Frappé P; Savall A							Vaccine perceived as risky and cervical smear encouraged.
Casciotti DM; Smith KC; Klassen AC	[68]	HPV	English		Newspaper articles	Tone analysis<br>Content analysis	Most articles were pro-vaccine in tone, prompted by scientific research or legislative activity<br><br>Concern peaked, as evidenced by fewer articles containing conflict before and after the peak
Catalan-Matamoros D; Elías C	[69]	General	Spanish		Newspaper articles	Content analysis<br>Tone analysis<br>Frame analysis	Sources related to government, professional association, and scientific companies most frequently used when discussing vaccines
Catalan-Matamoros D; Peñafiel-Saiz C	[78]	Childhood	Spanish		Newspapers	Thematic coding<br>Tone analysis	Inverse correlation between negative newspaper coverage and childhood vaccine uptake
Colón-Lopez V; Rivera-Figueroa V; Arroyo-Morales GO; Medina-Laabes DT; Soto-Abreu R; Rivera-Encarnación M; Díaz-Miranda OL; Ortiz AP; Wells KB; Vázquez-Otero C; Hull PC	[70]	HPV	Spanish		Newspapers	Content analysis	Earlier videos focused on discussion of HPV implementation policy; later videos focused on controversies around implementation<br><br>Neutral themes emerging: describing policy, information about HPV-related cancers, general information on HPV vaccine<br><br>Negative themes emerging: infringement to patient and parental autonomy, hesitancy from political sector, hesitancy from groups and coalitions
Court J; Carter SM; Attwell K;	[71]	Childhood	English		Australian print media	Frame analysis	Pejorative “anti-vax” term to negatively characterize non-vaccinating parents as: deviant

Author(s)	Ref. No.	Vaccine(s)	Language(s)	Theories	Platform(s)	Main method(s)	Findings
Leask J; Wiley K							others; ignorant; vulnerable and needing protection from anti-vaccination activists; lacking access to vaccination (opposed to unwilling)
Covolo L; Ceretti E; Passeri C; Boletti M; Gelatti U	[36]	Childhood	Italian		YouTube	Tone analysis	Pro-vaccine videos had higher views<br><br>Anti-vaccine videos more "liked" and shared
Criss S; Nguyen TT; Norton S; Virani I; Titherington E; Tillmanns EL; Kinnane C; Maiolo G; Kirby AB; Gee GC	[25]	COVID-19	English		Twitter	Content analysis<br>Sentiment analysis	Pro-vaccine themes: vaccine affirmation, advocacy through reproach, development and efficacy, news updates<br><br>Anti-vaccine themes: adverse reactions.<br><br>Misinformation themes: political misinformation, beliefs about immunity and protective behaviors, race extermination<br><br>Equity of access themes: overcoming medical racism, pointing out health disparities, facilitation of vaccine access
Das, M.K.; Singh, D.; Sharma, S.	[72]	Multiple	English		Online media news	Thematic coding<br>Sentiment analysis	Negative sentiment themes: adverse events, resistance to vaccines, and mention of measles-rubella vaccination campaign.
Diaz P; Reddy P; Ramasahayam R; Kuchakulla M; Ramasamy R	[63]	COVID-19	English		Google Trends (search queries)	Activity online	Increase in search terms on vaccine and infertility after CDC emergency approval
Donzelli G; Palomba G;	[37]	General	Italian		YouTube	Tone analysis<br>Engagement	Most videos uploaded were negative in tone

Author(s)	Ref. No.	Vaccine(s)	Language(s)	Theories	Platform(s)	Main method(s)	Findings
Federigi I; Aquino F; Cioni L; Verani M; Carducci A; Lopalco P							Number of videos increased annually
Fieselmann, J; Annac, K; Erdsiek, F; Yilmaz-Aslan, Y; Brzoska, P	[34]	COVID-19	German		Instagram<br>Youtube<br>Facebook	Thematic coding	Six categories: low perceived benefits, low perceived risk of contracting COVID-19, health concerns, lack of information, systemic mistrust, and spiritual/religious reasons.<br><br>Lack of information and spread of misinformation regarding COVID-19 vaccines<br><br>Not enough information: can be due to lack of information sensitive to needs of target group
Filice, E.; Dubé, E.; Graham, J.E.; MacDonald, N.E.; Bettinger, J.A.; Greyson, D.; MacDonald, S.; Driedger, S.M.; Kawchuk, G.; Meyer, S.B.	[136]	General	English		Professional guidelines and recommendations from complementary and alternative websites and academic literature	Discourse analysis	Academic literature themes: effectiveness, safety, empirical and ethical soundness, political justifiability, and compatibility with alternative medicine philosophy<br>Website themes: vaccine administration, counsel, education and marketing.
Gori, D.; Durazzi, F.; Montalti, M.; Di Valerio, Z.; Reno, C.; Fantini, M.P.; Remondini, D.	[31]	COVID-19	Italian		Twitter	Polarity analysis (sentiment on vaccines)	Most often retweeted users are moderately polarized

Author(s)	Ref. No.	Vaccine(s)	Language(s)	Theories	Platform(s)	Main method(s)	Findings
Griffith J; Marani H; Monkman H	[26]	COVID-19	English	Theoretical Domains Framework	Twitter	Thematic coding	Themes: concerns over safety, suspicion about political or economic forces driving pandemic and vaccine development, no knowledge on vaccine, antivaccine messages from authorities, lack of legal liability from vaccine companies.
Guidry JP; Carlyle K; Messner M; Jin Y	[64]	General	English		Pinterest	Thematic coding<br>Engagement<br>Content analysis	Most pins were anti-vaccine and were original posts (not re-pins)<br><br>Themes: side effects and safety of vaccines; conspiracy theories<br><br>Pro-vaccine pins received more engagement than anti-vaccine pins
Hou Z; Tong Y; Du F; Lu L; Zhao S; Yu K; Piatek SJ; Larson HJ; Lin L	[27]	COVID-19	Multiple	WHO's CCC Model	Twitter	Thematic analysis	Tweeters in London and New York: higher perceived risk of getting COVID-19, higher distrust in vaccine safety, distrust in government and experts, and widespread information and rumors. Focus on distribution and inequities.<br><br>Tweeters in Mumbai, Sao Paolo, and Beijing: worried about production and supply.<br><br>Overall, negative Tweets expressing lack of vaccine confidence and misinformation/rumors had more engagement.
Jamison AM; Broniatowski DA; Dredze M; Wood-Doughty Z; Khan D; Quinn SC	[39]	General	English		Facebook advertisement archives	Thematic coding	Median number of ads per buyer higher for anti-vaccine ads<br>Themes: vaccine harms (a majority)
Kalichman SC; Eaton LA;	[40]	COVID-19	English		Facebook posts	Thematic coding	Anti-vaccine groups discussed vaccines early on and had more influence than non-COVID-19 posts.

Author(s)	Ref. No.	Vaccine(s)	Language(s)	Theories	Platform(s)	Main method(s)	Findings
Earnshaw VA; Brousseau N							Themes: vaccine safety, and conspiracy theories around COVID-19
Karapetiantz P; Audeh B; Lillo-Le Louët A; Bousquet C	[56]	HPV	French		French web forums	Sentiment analysis	Majority of comments on HPV vaccines were negative
Keim-Malpass J; Mitchell EM; Sun E; Kennedy C	[28]	HPV	English		Twitter	Content analysis Sentiment analysis	Most Twitter posts written by lay consumers<br><br>51% positive comments
Kummervold PE; Schulz WS; Smout E; Fernandez-Luque L; Larson HJ	[73]	Ebola	English		Ghanaian online newspapers	Thematic coding	Critiques of vaccine trial: trials in secret, claims that trials would cause vaccine outbreak (testing on people gives as side effect), impropriety of incentives offered to participants (too large, most often complain too little)
Lahouati M; De Coucy A; Sarlangue J; Cazanave C	[35]	General	French		YouTube	Thematic coding, Stance coding,	Anti-vaccine websites had more views<br><br>Themes: side effects, pharmaceutical lobbying, and presence of adjuvants (safety concern in composition)
Larson HJ; Smith DM; Paterson P; Cumming M; Eckersberger E; Freifeld CC; Ghinai I; Jarrett C; Paushter L; Brownstein JS; Madoff LC	[51]	General	English		Media reports via HealthMap	Sentiment analysis Thematic coding	Negative reports topic areas: impacts and delivery of vaccine programs, beliefs awareness and perceptions, vaccine safety.

Author(s)	Ref. No.	Vaccine(s)	Language(s)	Theories	Platform(s)	Main method(s)	Findings
Loft LH; Pedersen EA; Jacobsen SU; Søborg B; Bigaard J	[45]	HPV	Danish		Facebook	Campaign evaluation<br>Sentiment analysis<br>Engagement online	Personal stories generated higher engagement rates
Luisi MLR	[47]	HPV	English	Social amplification of risk	Facebook	Analyzing online engagement	Risk-amplifying messages received greater reactions (comment, share counts)<br><br>Evidence of forward momentum of risk amplification and ripples
Luisi MLR	[46]	HPV	English	Health belief model	Facebook	Analyzing online engagement<br>Tone analysis	Barriers to HPV vaccination appeared more than benefits<br><br>Negative tone on vaccine more dominant than positive, and increased over time<br><br>Tone positive negatively correlated with barriers to HPV vaccination<br><br>Evidence for forward momentum of HPV infection susceptibility, severity.
Mahroum N; Watad A; Rosselli R; Brigo F; Chiesa V; Siri A; Ben-Ami Shor D; Martini M; Bragazzi NL; Adawi M	[65]	Influenza	Italian		Fluad-related web searches (Google)	Analyze activity online	Regions affected by Fluad-scandal had more scandal-related web searches.
Marchetti, F; Verazza, S; Brambilla, M; Restivo, V	[29]	Rotavirus	Italian		Facebook<br>Blogs<br>Twitter<br>YouTube	Sentiment analysis<br>Content analysis	Most sentiment was negative<br><br>Pediatricians and vaccination staff were influential health care professional

Author(s)	Ref. No.	Vaccine(s)	Language(s)	Theories	Platform(s)	Main method(s)	Findings
							Vaccine hesitant topics: fear of adverse events, concerns about vaccination schedule, COVID-19.
Meyer SB; Violette R; Aggarwal R; Simeoni M; MacDougall H; Waite N	[74]	Influenza	English		User comment threads, Canadian Broadcasting Corporation	Thematic coding Discourse analysis	Polarizing comments (for or against) more common<br><br>Forums mostly served as echo-chambers of affirmation of pre-existing beliefs.
Moran, M.B.; Lucas, M.; Everhart, K.; Morgan, A.; Prickett, E.	[52]	Childhood	English	Persuasion theory	Anti-vaccine websites	Content analysis Thematic coding	Anti-vaccine websites contain misinformation (on dangers, autism links, and brain injury)<br><br>Tactics: evidence and anecdotes<br><br>Themes: individual choice on vaccination, use of alternative medicine and homeopathy
Nugier A; Limousi F; Lydié N	[53]	General	French		French websites	Thematic coding	Themes: general concerns, infringement of individual freedoms, vaccinations as “unnatural”, overall negative benefit<br><br>Tactics: manipulation of science, use of shocking images, appeal to emotions via testimonies, perpetuation of general vaccination conspiracies<br><br>More negative keywords resulted in anti-vaccine websites
Odone A; Chiesa V; Ciorba V; Cella P; Pasquarella C; Signorelli C	[75]	Influenza	Italian		Italian newspapers	Thematic coding	When deaths reported, key words on the Fluad case spiked.
Olufowote JO	[76]	Polio	English		Nigerian newspapers	Content analysis Thematic coding	Main themes: western control and abuse through global organization, philanthropy of west is self-serving and malevolent, doubts about vaccine production and concern with side effects.

Author(s)	Ref. No.	Vaccine(s)	Language(s)	Theories	Platform(s)	Main method(s)	Findings
Orr D; Baram-Tsabari A; Landsman K	[41]	Polio	Hebrew		Online Hebrew Platforms, Facebook	Content analysis Thematic coding	Vaccine opposition themes: comments with individualistic perception, expressing safety of vaccine, expression of distrust in Israeli Ministry of Health, denying of Polio as disease.<br><br>Unmediated debate on Facebook platform.
Panatto D; Amicizia D; Arata L; Lai PL; Gasparini R	[57]	Influenza	Italian		Italian web pages	Sentiment, tone análisis	Negative information associated with less professional institutions<br><br>Negative information content focused on safety issues (link to diseases, to Gulf War Syndrome)
Pedersen EA; Loft LH; Jacobsen SU; Søborg B; Bigaard J	[48]	HPV	English		Facebook	Campaign evaluation	Personal stories created most positive dialogues<br><br>Increase in positive comments post-campaign
Ruiz JB; Bell RA	[61]	General	English		First-page Google search result	Content analysis	Perpetuation of many myths<br><br>Recommendation of no vaccination usually on a negative search term search
Sajjadi NB; Shepard S; Ottwell R; Murray K; Chronister J; Hartwell M; Vassar M	[62]	COVID-19	English		Google search activity	Content analysis	Most questions on vaccine safety and efficacy<br><br>Government sources provided most transparent information on vaccines
Sharon AJ; Yom-Tov E; Baram-Tsabari A	[60]	General	English	Epistemic Trust	Yahoo! Answers Facebook group	Thematic coding Sentiment analysis	Doctor opinions are more likely to be considered “best answer”

Author(s)	Ref. No.	Vaccine(s)	Language(s)	Theories	Platform(s)	Main method(s)	Findings
							Identity and stance of responding person affected credibility<br>Epistemic trust in mainstream science and medicine needs to be strong.
Shoup JA; Narwaney KJ; Wagner NM; Kraus CR; Gleason KS; Albright K; Glanz JM	[58]	Childhood	English		Vaccine social media website	Tone, sentiment, stance, coding	Publicly available sites more contentious and anti-vax<br><br>Expert-moderated sites more neutral and less inaccuracies
Stephenson, N.; Chaukra, S.; Katz, I.; Heywood, A.	[77]	Childhood	English		Newspapers	Frame analysis	Australian newspapers aligned with policy targets<br><br>Coverage of vaccine objectors intensified in quantity and negativity, portrayed as invisible and victims of being denied choice.
Sundstrom B; Cartmell KB; White AA; Well H; Pierce JY; Brandt HM	[30]	HPV	English		Facebook, Twitter	Campaign evaluation<br>Content analysis<br>Engagement analysis	Most pro- and anti-vaccine comments were dominated by personal stories.
Suppli CH; Hansen ND; Rasmussen M; Valentiner-Branth P; Krause TG; Mølbak K	[59]	HPV	Danish		Media coverage, Google search activity	Online activity<br>Media coverage	Decline in vaccination associated with adverse-event reporting in media
Toth C	[54]	General	English	Repertoires	Online content<br>Television (talk shows)<br>Interviews with parents	Content analysis	Common themes: distrust in vaccines, rejecting risks when it comes to children, vaccine ineffectiveness.

Author(s)	Ref. No.	Vaccine(s)	Language(s)	Theories	Platform(s)	Main method(s)	Findings
Tustin, J.L.; Crowcroft, N.S.; Gesink, D.; Johnson, I.; Keelan, J.; Lachapelle, B.	[42]	General	English		Facebook (comments)	Thematic coding, Sentiment analysis	Vaccine hesitancy themes: Distrust of pharma or government agencies, distrust of health care system providers, previous negative experiences with vaccination, and attitude around health.
Ward JK; Peretti-Watel P; Larson HJ; Raude J; Verger P	[134]	General	French English		Websites	Thematic coding	Recurring themes: safety and effectiveness of vaccine, alternative treatments, trust in authorities and medical recommendations, philosophical arguments against vaccination (religious, immoral, anti-utilitarian)<br><br>Questions on controversial topics returned more controversial pages
Ward, S.; Budarick, J.	[55]	General	English	Discursive legitimization; ideological squares	The Daily Telegraph	Campaign evaluation, Sentiment analysis	Initial intention of campaign to polarize to justify financial sanction against vaccine-hesitant parents<br><br>Generating concern through parental and motherhood figures better for communicating risk
Wawrzuta D; Jaworski M; Gotlib J; Panczyk M	[43]	COVID-19	Polish*		Facebook	Thematic coding	Twelve categories of negative arguments: seven universal; five COVID-19 specific:<br>lack of trust in government, vaccine does not exist or does not work, danger to health, COVID-19 does not exist, freedom of choice, conspiracy theories, existence of vaccines before pandemic, not properly tested, relation to past development of swine flu, created for profit, natural methods better than vaccines, lack of accountability for vaccine side-effects.
Wiyeh AB; Cooper S; Jaca A; Mavundza E; Ndwandwe	[44]	HPV	English		Facebook	Thematic coding	Mostly favorable towards vaccination<br><br>Hesitancy themes: reproductive health, low perceived risk of getting cervical cancer,

Author(s)	Ref. No.	Vaccine(s)	Language(s)	Theories	Platform(s)	Main method(s)	Findings
D; Wiysonge CS							questionable efficacy of vaccine, fear of girls being "used" for research<br><br>Pro-vaccination themes: knowing affected person, knowing about causes of cervical cancer, confidence in safety of vaccine, strong recommendation from WHO.
Wong, LP; Lin, Y; Alias, H; Bakar, SA; Zhao, Q; Hu, Z		COVID-19	English		Instagram<br>Facebook	Sentiment analysis<br>Thematic coding	Hesitancy themes: unprecedent production speed compromising safety, unknown long-term effects, vaccine composition concern, natural immunity from infection stronger.<br><br>Peer-reviewed publication favored as source of trust
Yeung, M.W.L.; Yau, A.H.Y.	[135]	General	Cantonese		Facebook	Content Analysis	More anti-vaccination sentiment<br><br>Mention of government always appeared with anti-vaccination statement<br><br>Recurring themes: personal experience, health professional image, vaccine research studies
Yiannakoulias N; Slavik CE; Chase M	[137]	Measles<br>Influenza	English		YouTube	Sentiment<br>Coding	Anti-immunization videos more liked<br><br>Anti-immunization content can be identified by a few words
Zhou F;<br>Zhang W;<br>Cai H;<br>Cao Y		HPV	Chinese		Weibo	Content Analysis	Vaccine lacks popularity<br><br>Topics focus on price<br><br>Little attention on safety and efficacy
