# Supplementary material for: Media Data and Vaccine Hesitancy: Scoping Review
Source: JMIR Infodemiology. 2022 Aug 10;2(2):e37300. doi: 10.2196/37300 (PMC9987198; doi:10.2196/37300)
Supplement: Multimedia Appendix 4 [file infodemiology_v2i2e37300_app4.pdf]

Author(s)	Ref. No.	Vaccine(s)	Language(s)	Theories	Platform(s)	Main method(s)	Findings
Ajovalasit S; Dorgali VM; Mazza A; d'Onofrio A; Manfredi P	[83]	General	Italian		Twitter	Machine classifier; Sentiment analysis	Interactions peaked in response to political events<br><br>Suggests evidence of disorientation among public.
Argyris YA; Monu K; Tan PN; Aarts C; Jiang F; Wiseley KA	[97]	General	English	Entman's message framing; Engagement-persuasion spectrum	Twitter	Machine classifier; Topic modeling	Higher engagement among antivax communities (more resilient)<br><br>Pro-vaccine advocates do not define problem but focus on criticizing antivax advocates.
Baines A; Ittefaq M; Abwao M	[122]	COVID-19	English	Echo chamber conceptual framework	Parler	Topic modeling	Vaccine refusal themes: side effects, population control through the vaccine, children vaccinated without parental consent, and comparison of other health issues with COVID-19<br><br>Users adopt different terms and hashtags to express beliefs
Bar-Lev S; Reichman S; Barnett-Itzhaki Z	[120]	Multiple: hepatitis b, diphtheria, tetanus, whooping cough, polio, pneumococcal, rotavirus, MMR	Hebrew; English		Facebook; Tapuz vaccination (forum)	Projection; Semantic network analysis	Higher volume of social media traffic, higher hesitancy.
Benis A; Chatsubi A; Levner E; Ashkenazi S	[101]	Influenza	English		Twitter	Semantic network analysis	Vaccine themes: clinical aspects of the vaccine (symptoms, reporting health conditions); protection and responsibility (of taking vaccine); politics (divergence of opinions from U.S. political leaders)<br><br>More tailored messages related to higher response, engagement, and pro-activeness of target population to get vaccinated.

Author(s)	Ref. No.	Vaccine(s)	Language(s)	Theories	Platform(s)	Main method(s)	Findings
Blankenship EB; Goff ME; Yin J; Tse ZTH; Fu KW; Liang H; Saroha N; Fung IC	[84]	General	English		Twitter	Topic modeling; Sentiment analysis	Anti-vax tweets had more retweets<br><br>Most anti-vaccine Tweets involved Twitter, YouTube, and Facebook links.<br><br>Most pro-vaccine Tweets are from Twitter, CDC, and Trap.it
Boucher JC; Cornelson K; Benham JL; Fullerton MM; Tang T; Constantinescu C; Mourali M; Oxoby RJ; Marshall DA; Hemmati H; Badami A; Hu J; Lang R	[102]	COVID-19	French; English	5c scale; Vaccine confidence index	Twitter	Semantic network analysis	Themes of vaccine hesitancy: concerns of safety and efficacy (of mRNA technology), freedom, and mistrust in institutions.<br>Concerns for freedom or mistrust of institutions (multinational corporations, or government)
Cafiero, F.; Guille-Escuret, P.; Ward, J.K.	[126]	General	French		Websites	Semantic network analysis	Vaccine critical activists are fragmented in belief<br><br>Some appear moderate but are deep in radical communities (use of “moderateness” for relatability)
Chen B; Zhang JM; Jiang Z; Shao J; Jiang T; Wang Z; Liu K; Tang S; Gu H; Jiang J	[113]	Kangtai Hepatitis V	Chinese		Weibo, online news, Baidu search	Sentiment analysis	Vaccine crisis raised public attention and negative sentiment online in China.

Author(s)	Ref. No.	Vaccine(s)	Language(s)	Theories	Platform(s)	Main method(s)	Findings
Cotfas LA; Delcea C; Gherai R	[85]	COVID-19	English		Twitter	Sentiment analysis; Topic modelling	Variation in Tweeting follows major events reporting (reactionary Tweeting)<br><br>Over time, Tweeting became less against vaccination
DeDominicis K; Buttenheim AM; Howa AC; Delamater PL; Salmon D; Omer SB; Klein NP	[131]	Vaccination requirements	English		Web pages, Facebook status, public group, newspaper article, blog posts, government reports, forum discussions. Snowball	Semantic network analysis; Topic modelling	Vaccine skeptics choose to argue about vaccine policy on ideological or legal grounds (using words like 'freedom' and choice' and a loss of 'human rights' or 'parental rights'); bring up historical human rights abuses; talk in macroscopic terms about America, government, constitutionality, legality; exhibit concern of government fraud and not having citizens' best interest.<br><br>Vaccine advocates focus on harms that vaccine hesitancy have on children, and dispute using scientific arguments.
Deiner MS; Fathy C; Kim J; Niemeyer K; Ramirez D; Ackley SF; Liu F; Lietman TM; Porco TC	[86]	General	English		Facebook; Twitter	Sentiment analysis	Pro-vaccination posts correlated with U.S. weekly reported case (suggesting episodic interest)<br><br>Anti-vaccination posts unrelated to presence of measles epidemic in U.S. (more constant messaging overall)
Du J; Xu J; Song HY; Tao C	[87]	HPV	English		Twitter	Machine classifier; Sentiment analysis	Negative tweets decrease then increase<br><br>Tweets containing worries on efficacy for HPV vaccines had relative significant decreasing trend.
Dunn AG; Surian D; Dalmazzo J; Rezazadegan D; Steffens M; Dyda A; Leask	[98]	HPV	English		Twitter	Topic modeling	Exposure to HPV related tweets explained more variance in state level HPV vaccine coverage than explained by socioeconomic factors<br><br>Suggest that states where negative opinions about

Author(s)	Ref. No.	Vaccine(s)	Language(s)	Theories	Platform(s)	Main method(s)	Findings
J; Coiera E; Dey A; Mandl KD							HPV vaccines are popularized by mainstream media will have lower coverage<br><br>News media may reflect, amplify, or influence vaccine acceptance
Dunn AG; Surian D; Dalmazzo J; Rezazadegan D; Steffens M; Dyda A; Leask J; Coiera E; Dey A; Mandl KD	[98]	General	English		Twitter	Machine classifier	Engagement with vaccine-critical information is concentrated in certain subgroups that are more engaged in the topic of vaccine hesitancy<br><br>Twitter users rarely share vaccine-related content posted by bots
Featherstone, J.D.; Ruiz, J.B.; Barnett, G.A.; Millam, B.J.	[103]	MMR, HPV, Tdap	English		Twitter	Semantic network analysis	Major themes: HPV vaccination is disease preventative, MMR vaccines links to autism, and rate of measles outbreak. concentrated Range of topics of vaccination on twitter were mostly negative.
Furini, M.	[121]	General	Italian	Product-harm crisis (marketing)	Facebook	Description	Crisis phases evident: number of posted messages increases significantly, and occur in relation to real world events<br>NoVax members tried to give authoritativeness to their statements, but backed on weak sources
Germani F; Biller-Andorno N	[104]	COVID-19	English		Twitter	Semantic network analysis	Anti-vaccination supporters: tweet less, but engage in more discussion; associated with a general belief on conspiracy theories and emotional behaviors; engaged in virtual community led by Donald Trump and other influencers<br><br>Pro-vaccination supporters more interested in: their own education and profession; produces more engaging content

Author(s)	Ref. No.	Vaccine(s)	Language(s)	Theories	Platform(s)	Main method(s)	Findings
							Emotional language can aid success of vaccination campaigns;
Gesualdo F; D'Ambrosio A; Agricola E; Russo L; Campagna I; Ferretti B; Pandolfi E; Cristoforetti M; Tozzi AE; Rizzo C	[88]	General	Italian		Twitter	Sentiment analysis	Vaccination discourse moved more towards promotional tweets.
Getman R; Helmi M; Roberts H; Yansane A; Cutler D; Seymour B	[127]	Childhood	English		Media Cloud	Semantic network analysis; Sentiment analysis	Vaccine hesitant community is a separate but robust network resistant to influence of pro-vax communities<br><br>Scientific evidence prominently used and misused within vaccine-hesitant community.
Guidry JPD; Austin LL; O'Donnell NH; Coman IA; Lovari A; Messner M	[110]	Influenza	English	Health belief model	Twitter	Description	Fewer vaccine-hesitant tweets appear to be present<br><br>Increase in barriers of flu vaccine uptake during peak season<br><br>Increasing mention of conspiracy theories during peak season
Gunaratne K; Coomes EA; Haghbayan H	[105]	General	English		Twitter	Semantic network analysis	Pro-vaccine tweet volumes larger than anti-vaccine and continue growing<br><br>Anti-vaccine community growing despite fewer tweets

Author(s)	Ref. No.	Vaccine(s)	Language(s)	Theories	Platform(s)	Main method(s)	Findings
							Ideological isolation due to minimal inter-communication
Guntuku SC; Buttenheim AM; Sherman G; Merchant RM	[99]	COVID-19	English		Twitter	Topic modeling	Discourse changes around different communities over time: urban/suburban about distribution; college towns about in-clinic vaccination; evangelical hubs thanking God; Hispanic centers around safety of food and water; African American South about trust, hesitancy and history; 'gray' America talking about federal government failures.<br><br>Inform targeted messaging and mitigation strategies needed
Hu D; Martin C; Dredze M; Broniatowski DA	[115]	General	Chinese		Weibo	Topic modeling	Expression of distrust increased during and immediately after scandal (episodic)<br><br>Self-report of vaccination shifts from positive endorsement to concerns about harms.
Hu T; Wang S; Luo W; Zhang M; Huang X; Yan Y; Liu R; Ly K; Kacker V; She B; Li Z	[89]	COVID-19	English		Twitter	Sentiment analysis; Topic modeling	Critical social or international events or announcements by poiltical leaders impact public opinion (response to Kamala Harris, Bill Gates, Donald Trump)<br><br>Conspiracy theories led to sharp decline in sentiment scores<br><br>Different states demonstrated various trends in sentimental and emotional scores
Jiang, X.; Su, M.-H.; Hwang, J.; Lian, R.; Brauer, M.;	[100]	COVID-19	English		Twitter	Semantic network analysis; Topic modeling	Twitter users expressing conservative ideology express less favorable vaccine-related sentiments, and talk more about side effects, distrust of medical professional, and conspiracy theories

Author(s)	Ref. No.	Vaccine(s)	Language(s)	Theories	Platform(s)	Main method(s)	Findings
Kim, S.; Shah, D.							Political alignment: liberals less distrusting of medical professionals<br>different topics of focus between the two camps. Also, liberal focus on development and role in ending pandemic; and conservative users discuss potential misconduct.
Kang GJ; Ewing-Nelson SR; Mackey L; Schlitt JT; Marathe A; Abbas KM; Swarup S	[124]	General	English		Websites (via Twitter Chattergrabber)	Semantic network analysis; Sentiment analysis	Positive vaccine sentiment networks are more cohesive relative to negative vaccine sentiment networks<br><br>Positive networks centered around parents. Negative networks centered around children<br><br>Framing of negative networks around skepticism and distrust of government organizations that support vaccination
Klimiuk K; Czoska A; Biernacka K; Balwicki Å•	[117]	Childhood	Polish		Facebook	Sentiment analysis	Large amount of content in: conspiracy theories, misinformation, safety and effectiveness of vaccination, noncompliance with civil rights, morality/religious beliefs, and alternative medicine<br><br>Possible indication that authors are characterized by a lack of trust in scientific achievements
Kummervold PE; Martin S; Dada S; Kilich E; Denny C; Paterson P; Larson HJ	[111]	Any during pregnancy	English		Twitter	Description	Can achieve close to same accuracy in categorizing tweets using machine learning as opposed to humans.
Lee H; Noh EB; Park SJ; Nam HK; Lee	[128]	COVID-19	Korean		Naver; Instagram;	Sentiment analysis	Negative perception of vaccines, with more for Astrazeneca over Pfizer<br><br>Many people focused on side effects.

Author(s)	Ref. No.	Vaccine(s)	Language(s)	Theories	Platform(s)	Main method(s)	Findings
TH; Lee GR; Nam EW							
Liew TM; Lee CS	[82]	COVID-19	English	COM-B (capability, opportunity, motivation components of behavior)	Twitter	Sentiment analysis	Six overarching themes related to vaccination:<br>1. Emotional reactions<br>2. Public concerns<br>3. Discussions about news items<br>4. Public health communication<br>5. Discussions about approaches to COVID-19 vaccination drive<br>6. Discussion about distribution of COVID-19 vaccine
Luo C; Ji K; Tang Y; Du Z	[133]	COVID-19	Chinese		Zhihu	Sentiment analysis; Topic modelling	Laypersons are more prevailing in vaccine discussion, but with less accurate risk perception<br><br>Professionals' answers are longer than laypersons, and focus more on arrangement and urgent approval<br><br>Both groups focus on: adverse reactions, worldwide symptoms, effectiveness against mutants, and contribution of vaccination towards global disease prevention.
Lutkenhaus RO; Jansz J; Bouman MPA	[106]	General	Dutch		Twitter	Semantic network analysis	Debate on vaccination spearheaded by writers and journalists and influenced by pro-health and anti-health establishment<br><br>Info shared by health community does not spill over into larger community, but anti-health communities spill into other communities
Lyu H; Wang J; Wu W; Duong V; Zhang X; Dye TD; Luo J	[109]	COVID-19	English		Twitter	Feature extraction and modelling	Women more likely to hold hesitant opinions<br><br>Older people are more pro-vax

Author(s)	Ref. No.	Vaccine(s)	Language(s)	Theories	Platform(s)	Main method(s)	Findings
							Lower-income group more likely to hold polarized opinion<br><br>Religious people more likely to be polarized<br><br>Political diversion indicates a divided opinion about potential covid-19 vaccines<br><br>People living in suburban or rural areas are more likely to be anti-vaccine<br><br>Personal experience with COVID-19 and the county level pandemic severity perception shape the opinion
Lyu JC; Han EL; Luli GK	[90]	COVID-19	English		Twitter	Sentiment analysis; Topic modelling	Five themes arise: opinions around vaccination, knowledge about vaccines, vaccines as global issue, vaccine administration, and progress on vaccine development and authorization<br><br>Sentiment generally increasing positive<br><br>Discussions are driven by major events
Marcec R; Likic R	[107]	COVID-19	English		Twitter	Semantic network analysis	Sentiments on Pfizer/BioNTech and Moderna were stable and positive, Astrazeneca was decreasing in positivity<br><br>Pfizer and Moderna experienced periods of spikes;
Martin S; Kilich E; Dada S; Kummervold PE; Denny C;	[81]	Maternal	Multiple		Twitter; forums; blogs;	Semantic network analysis; Sentiment analysis; Description	Main topics around discussion of safety<br><br>Common topics: promotion of vaccination, involvement of pregnant women in vaccine research, transparency of institutions

Author(s)	Ref. No.	Vaccine(s)	Language(s)	Theories	Platform(s)	Main method(s)	Findings
Paterson P; Larson HJ							
Melton CA; Olusanya OA; Ammar N; Shaban-Nejad A	[123]	COVID-19	English		Reddit	Sentiment analysis	Overall positive sentiments towards vaccination which remained static<br><br>Reddit community mainly focused on side effects rather than conspiracy theories
Monselise M; Chang CH; Ferreira G; Yang R; Yang CC	[91]	COVID-19	English		Twitter	Sentiment analysis; Topic modeling	Administration and access to vaccines major concerns<br><br>Peaks of events were impacted by events reported in news and spread through social media<br><br>Main reaction on social media was fear<br><br>Make recommendations against WHO guidelines.
Okuhara T; Ishikawa H; Okada M; Kato M; Kiuchi T	[125]	Influenza	Japanese		Websites via Google Japan and Yahoo! Japan	Topic modeling	1/3 of content were on anti-websites, written by laypersons<br><br>Anti-vaccination websites focused on bad sides of vaccine industry<br><br>Pro vaccination websites: focus on efficacy rate, and effect of vaccination<br><br>Lack of confidence in effectiveness is one reason for not being vaccinated<br><br>Side effects was 2nd most frequent topic<br><br>Vaccine recommendations for elderly and patients with underlying disease were referred to on pro

Author(s)	Ref. No.	Vaccine(s)	Language(s)	Theories	Platform(s)	Main method(s)	Findings
							websites since target group under Preventive Vaccination Law
Pananos AD; Bury TM; Wang C; Schonfeld J; Mohanty SP; Nyhan B; Salathé M; Bauch CT	[108]	MMR	English	Theory of critical transitions	Twitter	Projection	Population vaccination behavior near disease elimination threshold has a critical point.
Piedrahita-Valdés H; Piedrahita-Castillo D; Bermejo-Higuera J; Guillem-Saiz P; Bermejo-Higuera JR; Guillem-Saiz J; Sicilia-Montalvo JA; Machío-Regidor F	[92]	General	English; Spanish		Twitter	Sentiment analysis	Neutral tweets decreased; positive and negative increased<br><br>Negative tweets most common in Netherlands, Canada, Japan, and United States<br><br>Most Twitter users has a homogeneous polarization discourse
Porreca A; Scozzari F; Di Nicola M	[129]	Childhood	Italian		YouTube; Google Trends	Semantic network analysis; Sentiment analysis	After communication campaign, and aggressive push my medical doctors, sentiment changed from negative to positive.
Powell GA; Zinszer K; Verma A; Bahk C; Madoff L;	[130]	General	English		Multiple via Vaccine Sentimeter	Description; Sentiment analysis	Negative comments mostly about vaccine safety<br><br>High geographical variation in media content

Author(s)	Ref. No.	Vaccine(s)	Language(s)	Theories	Platform(s)	Main method(s)	Findings
Brownstein J; Buckeridge D							
Schmidt AL; Zollo F; Scala A; Betsch C; Quattrociocchi W	[118]	General	English		Facebook	Description; Semantic network analysis	Vaccine content consumption is echo-chambered; polarization increases and creates well-segregated communities
Shim JG; Ryu KH; Lee SH; Cho EA; Lee YJ; Ahn JH	[93]	COVID-19	Korean		Twitter	Sentiment analysis; Topic modeling	Vaccine hesitation topics: development of vaccine, efficacy of vaccination, adverse reactions, medical associations' responses<br><br>Similar ratio of positive to negative tweets before and immediately after vaccination.
Tavoschi L; Quattrone F; D'Andrea E; Ducange P; Vabanesi M; Marcelloni F; Lopalco PL	[94]	General	Italian		Twitter	Sentiment analysis	Vaccine-related events influence number of and opinion polarity of tweets (e.g. mandatory immunization produced increased number of tweets)
Tomaszewski T; Morales A; Lourentzou I; Caskey R; Liu B; Schwartz A; Chin J	[112]	HPV	English		Twitter	Machine classifier	False information messages contain mostly loss-framed messages focusing on potential risk, and use a wider range of vocabulary<br><br>True information messages contained both gain- and loss-framed messages but used fewer vocab and limited vocabulary.
Wang Z; Yin Z; Argyris YA	[132]	General			Instagram	Image analysis	Possible to detect antivaccine messages on Instagram platform
Yan C; Law M; Nguyen S;	[95]	COVID-19	English		Reddit; Twitter	Sentiment analysis;	Engagement on Reddit correlates with daily new COVID-19 cases

Author(s)	Ref. No.	Vaccine(s)	Language(s)	Theories	Platform(s)	Main method(s)	Findings
Cheung J; Kong J						Topic modeling	Themes on Reddit related to vaccines: vaccine scarcity, vaccine uptake<br><br>Increase in vaccine-related discussion correlated with number vaccinated<br><br>Vaccine-related comments more positive (observed for many cities)
Yin, F.; Wu, Z.; Xia, X.; Ji, M.; Wang, Y.; Hu, Z.	[116]	COVID-19	Chinese		Weibo	Projection	Vaccine acceptance based on price and side effect<br><br>Positive sentiment about side effects over time collective<br><br>More debate around inactivated vaccines
Yousefinaghani S; Dara R; Mubareka S; Papadopoulos A; Sharif S	[96]	COVID-19	English		Twitter	Sentiment analysis	More positive tweets had higher engagements, but overall more anti-vaccine tweets<br><br>Vaccine opposition content were partly twitter bots or other anti-vaccination advocates<br><br>Negative topics: fear and frustration, disappointment, anger, politics
Zhang J; Xue H; Calabrese C; Chen H; Dang JHT	[119]	HPV	English		Facebook	Sentiment analysis; Topic analysis	Low vaccination coverage counties had fewer posts<br><br>Comments on HPV vaccine exhibited more positive emotion<br><br>Themes of posts: awareness and screening of HPV and cervical cancer, STI testing, information, and calls to action

Author(s)	Ref. No.	Vaccine(s)	Language(s)	Theories	Platform(s)	Main method(s)	Findings
							Promotion of HPV vaccine is limited in frequency and content diversity (not well-tailored)
Zhang Z; Feng G; Xu J; Zhang Y; Li J; Huang J; Akinwunmi B; Zhang CJP; Ming WK	[114]	COVID-19	Chinese		Weibo	Sentiment analysis	Women experience and express more intense emotion than men<br><br>Female users more likely to seek emotional support<br><br>Emotional tendencies of public respond to adverse event with decline in positive sentiment
